# Association between Human Papillomavirus and *Chlamydia trachomatis* genital infections in male partners of infertile couples

**DOI:** 10.1038/s41598-021-99279-9

**Published:** 2021-10-07

**Authors:** Carolina Olivera, Jessica P. Mosmann, Daniela A. Paira, Rosa I. Molina, Andrea D. Tissera, Rubén D. Motrich, Cecilia G. Cuffini, Virginia E. Rivero

**Affiliations:** 1 Centro de Investigaciones en Bioquímica Clínica E Inmunología, CIBICI-CONICET, Facultad de Ciencias Químicas. Universidad Nacional de Córdoba. Haya de La Torre Y Medina Allende, Ciudad Universitaria, X5016HUA Córdoba, Argentina; 2 Departamento de Bioquímica Clínica, Facultad de Ciencias Químicas, Universidad Nacional de Córdoba, Córdoba, Argentina; 3 Laboratorio de Andrología Y Reproducción, Córdoba, Argentina; 4 Instituto de Virología “Dr. José M. Vanella”, Facultad de Ciencias Médicas, Universidad Nacional de Córdoba, Córdoba, Argentina; 5 Consejo Nacional de Investigaciones Científicas Y Técnicas (CONICET), Buenos Aires, Argentina

**Keywords:** Immunology, Microbiology, Medical research, Urology

## Abstract

The prevalence of HPV infection and its relationship with other sexually transmitted infections was analyzed in a cohort of 117 male partners of infertile couples from Cordoba, Argentina. Semen samples and urethral swabs were obtained and the infection with HPV, *Chlamydia trachomatis*, HSV1, HSV2, *Mycoplasma hominis* and *Ureaplasma urealyticum* was analyzed. A prevalence of HPV infection of 27.4% was found. Interestingly, infections by exclusively low risk HPV genotypes or high/intermediate risk HPV genotypes were present in 64.5% and 22.6% of cases, respectively. Low risk-HPV6 was the most frequently detected genotype. Remarkably, HPV and *C. trachomatis* infections were significantly associated to each other (OR: 11.55, 95% CI 1.14–117.06). No significant differences in sperm quality were found between HPV-positive and HPV-negative patients indicating that HPV male urogenital infection does not impair sperm quality. Our results show a high prevalence of HPV urogenital infection among male partners of infertile couples, and that HPV and *C. trachomatis* infections are reciprocal risk factors of their co-infection. Moreover, our results suggest that men constitute a reservoir for continued transmission of *C. trachomatis* and HPV to women highlighting the need for routine screening for these two pathogens in male partners of infertile couples.

## Introduction

Sexually active couples who cannot achieve pregnancy after one year or more of regular unprotected sexual intercourse are currently considered infertile. Reported data indicate that approximately 10 to 20% of couples in reproductive age have fertility problems. Among the latter, male factor is involved in up to 50% of cases^1,2^. Several conditions have been associated with male factor infertility such as abnormalities of urogenital tract, malignancies, endocrine disorders, immune conditions and sexually transmitted infections (STI). Nevertheless, infertility still remains idiopathic in an important proportion of men^1,2^.

STI have been proposed to induce male infertility through multiple pathophysiological mechanisms^2–4^. STI in men may cause urethritis, prostatitis, vesiculitis, epididymitis and orchitis^3,4^. Although manifest signs and symptoms are present in many STI, in others the course of the infection is asymptomatic leading to untreated chronic infections that could impair fertility^3,4^. Certainly, available evidence indicates a higher prevalence of sexually transmitted bacterial (e.g., *Chlamydia trachomatis*, *Mycoplasma* spp.,* Ureaplasma* spp.) and viral (e.g., Herpes simplex virus, Human Papillomavirus) infections in infertile men compared with fertile men^4–6^.

Human Papillomavirus (HPV) is one of the most common sexually transmitted viral infections in males and females worldwide^7,8^. Low risk (LR) HPV genotypes can cause genital warts and respiratory papillomatosis, whereas persistent infection with high risk types (HR) can promote malignant transformations of epithelial cells in the cervix, but also in the vagina, vulva, anus, penis, mouth and throat^8–10^. The natural history of HPV infections in men is less known than in women^11^. Some studies suggest that male urogenital HPV infections usually clears spontaneously over time and only persists in a small fraction of the male population^3,12,13^. HPV may be detected anywhere in the male reproductive tract of infected patients such as external genitalia (scrotum, glans, penile shaft), anal region, perineum, testis, epididymis, vas deferens, urethra and semen^3,14^. HPV prevalence and load significantly varies in samples from different anatomic sites, showing higher proportions in external genitalia epithelial specimens than in semen^14–16^.

Despite the increasing evidence of HPV prevalence in semen, the worldwide distribution of HPV genotypes and their risk for male infertility remain inconclusive. Two meta-analysis and systematic reviews of HPV infection in semen showed an overall higher prevalence in men undergoing initial evaluation and/or treatment for infertility) in comparison with men from general populations^17,18^. The impact of HPV infection on sperm quality is also a controversial matter^17–21^. On the one hand, some studies have indicated a significant association between seminal HPV infection and low sperm quality, mainly impairing sperm motility^22–24^, while other studies have found no association^15,25^. Interestingly, only a few of those studies analyzed the presence of other putative concomitant STI besides HPV infection that could also affect sperm quality^19,25^.

The aim of the present study was to evaluate the burden of HPV infection and its relationship with other STI in a cohort of male partners of infertile couples from Cordoba, Argentina. Prevalence and genotype distribution of HPV infection, sperm quality and the association of HPV infection with other STI such as *Chlamydia trachomatis*, *Mycoplasma hominis*, *Ureaplasma urealyticum* and Herpes simplex virus were analyzed in this study.

## Results

### Prevalence of genital HPV infection and most common HPV genotypes in male partners of infertile couples

One hundred seventeen male partners of infertile couples were enrolled in our study. Characteristics of patients and their female partners segregated according to HPV infection status are shown in Table 1. HPV detection was performed by PCR on combined urethral swab and semen specimens to increase the chances of detecting male genital HPV infection^26^. The study population age ranged from 25 to 63 years, while their female partner´s age ranged from 22 to 46 years. Primary and secondary infertility was present in 81.2% and 18.8% of cases, respectively, while the mean time being unable to get a clinical pregnancy was 2.3 years. No significant differences in the mean patient age or female partner age, type of infertility and the time being unable to get a clinical pregnancy in their female partners, were found between HPV + and HPV- male patients (Table 1). Moreover, no significant associations between smoking habits of patients with HPV infection was found (*p* = 0.965, Pearson's chi-square test) (Table 1). An overall prevalence of HPV infection of 27.4% (32/117) was found in the population of male partners of infertile couples under study (Table 1). Regarding HPV genotypes, they could be determined in 31 of the 32 positive samples (see Supplementary Figure S1 online); it was impossible to assess the infecting HPV genotype in 1 patient (3.1%, unclassified) due to insufficient amount of sample (Table 2). Interestingly, infections by single HPV genotypes were detected in 84.4% (27 out of 32) of cases, whereas multiple infections were composed of no more than two HPV genotypes and detected in only 12.5% (4 out of 32) of cases (Table 2). When analyzing the identified HPV genotypes, infections by exclusively low risk HPV (LR-HPV) genotypes were found in 64.5% (20 out of 31) of cases, whereas infections only by high or intermediate risk HPV (HIR-HPV) genotypes were present in 22.6% (7 out of 31) of cases. Remarkably, LR-HPV6 genotype was by far the most frequently detected genotype, accounting for 71.9% (23/32) of all HPV infections, either single or multiple infections. Other LR-HPV genotypes such as HPV11 and HPV72 were found in only two different samples (HPV11 as a single infection and HPV72 co-infecting with LR-HPV6) (Table 2). A total of 10 HIR-HPV cases were found, of which 7 were single infections and 3 multiple infections, being HPV16, HPV18, HPV53 and HPV31 the most prevalent detected HIR genotypes (Table 2).

**Table 1:** Patient characteristics and descriptive statistics of the whole cohort segregated according to HPV status.

	Overall	HPV-	HPV +	*p value*
Number of individuals. n (%)	117 (100)	85 (72.6)	32 (27.4)	
**Age (years)**				*0.717*
Mean	37.4	37.4	37.3	
Range	25–63	25–54	27–63	
**Female partner´s age (years)**				*0.610*
Mean	34.4	34.5	34.3	
Range	22–46	22–46	29–43	
**Type of infertility. n (%)**				*0.638*
Primary	95 (81.2)	68 (80)	27 (84.3)	
Secondary	22 (18.8)	17 (20)	5 (15.6)	
**Time being unable to get a clinical pregnancy (years)**				*0.124*
Mean	2.3	2.2	2.8	
Range	1–10	1–10	1–10	
**Smoking status. n (%)**				*0.965*
No smokers	85 (72.6)	62 (72.9)	23 (71.9)	
Active smokers	32 (27.4)	23 (27.1)	9 (28.1)	

*HPV* Human Papillomavirus. Comparisons were performed between HPV positive and HPV negative groups, *p*-values calculated using the Mann–Whitney test in the case of age, female partner´s age and time being unable to get a clinical pregnancy, and Chi-square test in the case of type of infertility and smoking status. Differences were considered statistically significant when *p* < 0.05.

**Table 2:** Characterization of HPV infection in positive samples.

HPV prevalence	Cases (n)	Percentage (%)
Single HPV genotype infection	27/32	84.4
Multiple HPV genotype infection	4/32	12.5
Unclassified HPV genotype(s)	1/32	3.1
**Exclusively LR-HPV genotype(s)**	20/31	64.5
HPV6	19/31	61.3
HPV11	1/31	3.2
**Exclusively HIR-HPV genotype(s)**	7/31	22.6
HPV53	2/31	6.5
HPV31	2/31	6.5
HPV16	1/31	3.2
HPV18	1/31	3.2
HPV82	1/31	3.2
**LR and HIR-HPV genotype(s)**	3/31	9.7
HPV6 + HPV16	2/31	6.5
HPV6 + HPV18	1/31	3.2
**Two LR-HPV genotype(s)**	1/31	3.2
HPV6 + HPV72	1/31	3.2

*HPV* Human Papillomavirus, *HIR* High or Intermediate Risk, *LR* Low Risk. *Unclassified HPV genotype(s)* This includes a single patient´s sample whose genotype could not be classified due to insufficient sample.

### Co-infection of HPV and other pathogens

Number of positive cases and prevalence of *C. trachomatis*, *M. hominis, U. urealyticum,* HSV1, HSV2 and common bacteria (which include *E. coli, E. faecalis, P. mirabilis*) infection within the total patient population and within HPV-positive and HPV-negative patients are shown in Table 3. Four *C. trachomatis* (4/5), five *M. hominis* (5/24), seven *U. urealyticum* (7/28), three HSV1 (3/12), eight HSV2 (8/28) and two common bacterial infections (*E. Faecalis*) (2/17) were found in HPV positive men (Table 3). It is important to highlight that 8 out of 10 of the HIR-HPV genotypes detected in our study were co-infecting with other common bacterial or viral pathogens, while the two remaining HIR-HPV infection cases were within the group of patients without co-infections (data not shown).

**Table 3:** Prevalence of other sexually transmitted infections in HPV-positive and HPV- negative male partners of infertile couples.

Pathogen	Total cases (n)	Total Percentage (%)	HPV + (n = 32)		HPV- (n = 85)	
			n	Percentage (%)	n	Percentage (%)
HSV1	12	10.3	3/12	25.0	9/12	75.0
HSV2	28	23.9	8/28	28.6	20/28	71.4
*C. trachomatis*	5	4.3	4/5	80.0	1/5	20.0
*M. hominis*	24	20.5	5/24	20.8	19/24	79.2
*U. urealyticum*	28	23.9	7/28	25.0	21/28	75.0
**Common bacteria**						
*E. faecalis*	13	11.1	2/13	15.4	11/13	84.6
*E. coli*	3	2.6	0/3	0	3/3	100
*P. mirabilis*	1	0.9	0/1	0	1/1	100

*HPV* Human Papillomavirus, *HSV1* Herpes Simplex Virus type 1, *HSV2* Herpes Simplex Virus type 2.

**Figure 1:**
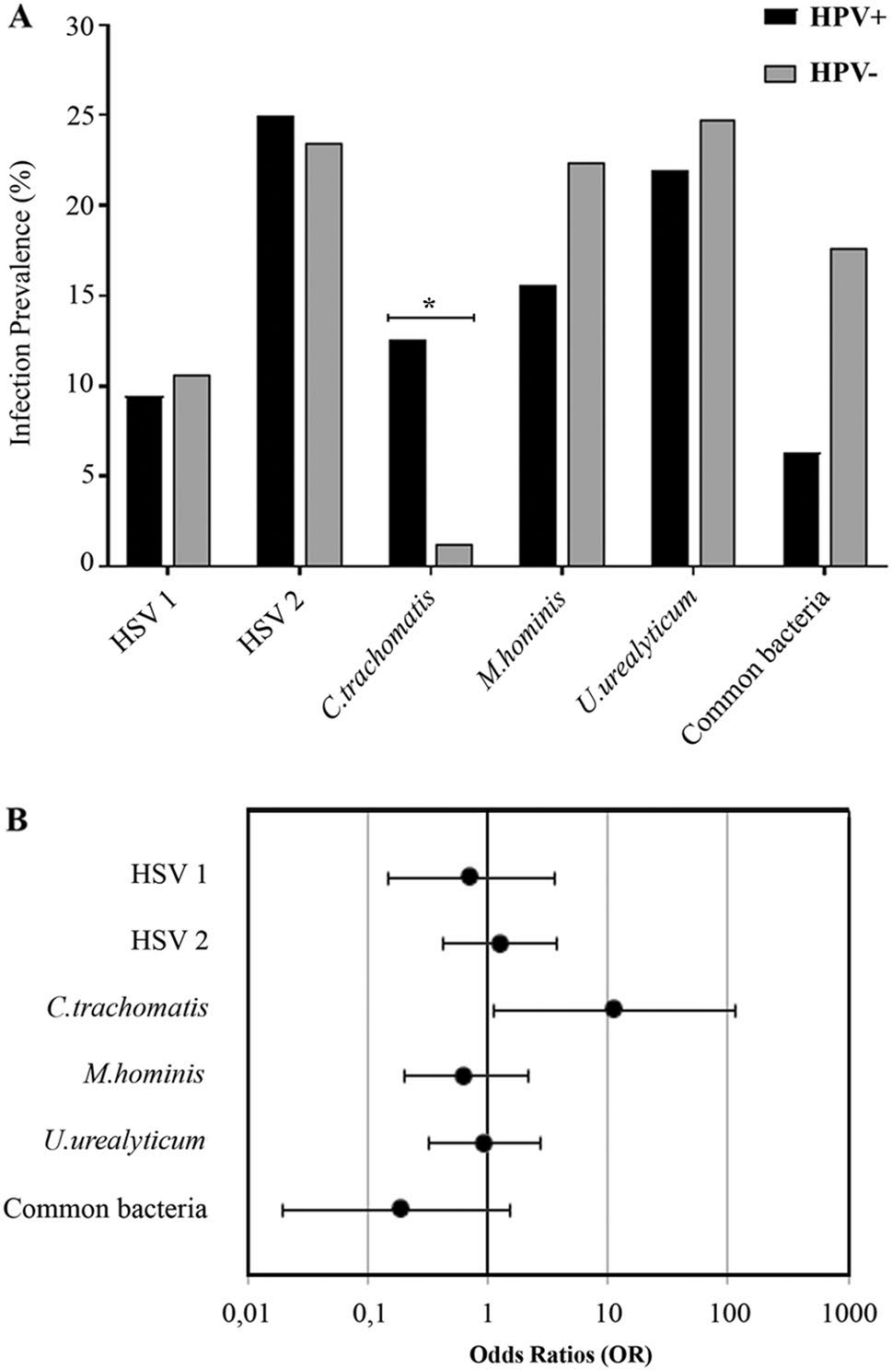
Total prevalence of infections by other common uropathogens and their association with HPV infection. (**A**) Total prevalence of HSV1, HSV2, *Chlamydia trachomatis*, *Mycoplasma hominis*, *Ureaplasma urealyticum* and other common uropathogenic bacterial (*Escherichia coli*, *Enterococcus faecalis* and *Proteus mirabilis*) infections were analyzed in HPV-positive (black bars) or HPV-negative (grey bars) groups. Pearson's Chi-squared test or Fisher's Exact Test test were performed;* p* values * < 0.05. (**B**) Forest plot contrasting the odds ratios (OR) and 95% confidence intervals for the analysis of the association between the previously mentioned infections and HPV infection. OR = 1 exposure does not affect odds of outcome, OR < 1 exposure associated with lower odds of outcome, OR > 1 exposure associated with higher odds of outcome.

On the one hand, when comparing frequencies of infection by *C. trachomatis*, *M. hominis*, *U. urealyticum*, HSV1, HSV2 and other common bacteria in HPV-positive and HPV-negative men (Fig. 1A), it was found that *C. trachomatis* infection was significantly associated with HPV infection (*p* = 0.019, Fisher's Exact Test), while no significant differences were observed when comparing the presence of *M. hominis*, *U. urealyticum*, HSV1, HSV2 and other common bacteria between HPV + and HPV− group.

Additionally, to assess whether any of the factors under study could be associated with HPV infection, bivariate and multivariate analyses were performed. Only subjects with all the pathogens analyzed were included in these analyses (n = 101). The estimated odds ratios (OR) for HPV and different pathogens analyzed in the bivariate analysis are shown in Fig. 1B. Remarkably, an OR of 11.55 (95% CI: 1.14–117.06) was found between *C. trachomatis* and HPV infections. On the contrary, other tested infections were not associated with HPV infection, with the following OR values: *M. hominis* (0.65, 95% CI 0.20–2.16), *U. urealyticum* (0.96, 95% CI 0.33–2.76), HSV1 (0.73, 95% CI 0.15–3.65), HSV2 (1.27, 95% CI 0.43–3.71), common bacteria (0.19, 95% CI 0.02–1.53) (Fig. 1B). When a multivariate analysis was carried out, *C. trachomatis* was again the only pathogen significantly associated with HPV. Either patient´s or his partner age, type of infertility, time being unable to get a clinical pregnancy, smoking status and the other co-infections analyzed were not correlated with HPV infection (see Supplementary Table S1 online). These results indicate that patients who are positive for *C. trachomatis* are nearly 12 times more likely to have an HPV infection than *C. trachomatis*-negative patients.

### HPV with or without co-infection impact on semen quality

Sperm quality from HPV-positive and HPV-negative patients was also analyzed. No significant differences were found in any of the sperm quality parameters (volume, sperm concentration, viability, motility and normal morphology) assessed between HPV-positive and HPV-negative patients (Table 4). Although peroxidase-positive cell concentrations were above normal values in both groups, values were similar in HPV-positive and HPV-negative patients (Table 4). Moreover, no significant differences in semen quality were found between HIR-HPV, LR-HPV and HPV-negative groups (data not shown). Semen quality analysis was also performed in HPV-positive and HPV-negative patients with or without co-infection by the other uropathogens assessed (Table 4). Patients were classified into four groups according to the presence or absence of HPV with or without co-infection with the other uropathogens: a group of patients in whom only HPV infection was detected (HPV + Coinf-, n = 12), a group of patients positive for HPV and for at least one of the other uropathogens (HPV + Coinf + , n = 20), a group of patients negative for all uropathogens assessed (HPV-Coinf-, n = 35), and a group of patients negative for HPV infection but positive for the infection with one or more of the other uropathogens (HPV-Coinf + , n = 50). No significant differences were found among groups when volume, sperm concentration, viability, motility, normal morphology and peroxidase-positive cell levels were analyzed (Table 4).

**Table 4:** Semen quality analysis of HPV-positive and HPV-negative patients with or without co- infections by other pathogens.

Sperm parameters	HPV + (n = 32)	HPV- (n = 85)	p* value	HPV + COINF- (n = 12)	HPV- COINF- (n = 35)	HPV + COINF + (n = 20)	HPV-COINF + (n = 50)	*p#* value
Volume (ml/ejaculate)	3.17 ± 1.75	3.02 ± 1.39	**0.803**	3.64 ± 1.29	2.85 ± 1.52	2.92 ± 1.93	3.14 ± 1.30	**0.452**
Sperm concentration (10^6^/mL)	48.98 ± 36.93	51.95 ± 40.05	**0.847**	56.06 ± 40.50	52.55 ± 43.86	39.02 ± 24.56	51.54 ± 37.61	**0.746**
Viability (%)	83.81 ± 8.58	82.58 ± 8.59	**0.384**	85 ± 5.33	83.37 ± 8.61	84.42 ± 8.46	82.02 ± 8.63	**0.522**
Total motility (PR + NP, %)	44.69 ± 19.84	48.65 ± 18.08	**0.306**	48.5 ± 21.11	49.29 ± 17.90	42.4 ± 19.23	48.20 ± 18.37	**0.592**
Progressive motility (PR, %)	43.16 ± 20.62	46.60 ± 18.76	**0.391**	47.67 ± 21.56	47.37 ± 18.54	40.45 ± 20.1	46.06 ± 19.08	**0.601**
Normal morphology (%)	6.23 ± 4.22	6.53 ± 3.88	**0.752**	7.44 ± 4.48	6.06 ± 2.97	5.59 ± 4.08	6.83 ± 4.38	**0.601**
Peroxidase-positive cells (10^5^/ml)	3.85 ± 4.51	6.67 ± 1.30	**0.979**	1.35 ± 1.45	4.91 ± 7.33	5.07 ± 5.14	6.35 ± 1.17	**0.185**

*HPV* Human Papillomavirus. All data are presented as mean ± SD. Total motility is the sum of Progressive motility (PR) and Non-Progressive motility (NP, data not shown). *HPV + * All HPV positive patients. *HPV-:* All HPV negative patients. *Comparisons were performed between HPV positive and HPV negative groups, *p*-values calculated using the Mann–Whitney test. Differences were considered statistically significant when *p* < 0.05. *HPV + COINF-* HPV positive patients without other infection. *HPV-COINF*HPV negative patients without other infection. *HPV + COINF + * HPV positive patients with a co-infection by at least one of the following pathogens analyzed: HSV1, HSV2, *C. trachomatis*, *M. hominis*, *U. urealyticum*, *E. faecalis*, *E. coli*, *P. mirabilis*. *HPV-COINF + * Only one or more of the other pathogens was detected and were negative for HPV. #Comparisons were performed between the four groups, *p*-values calculated using Kruskal–Wallis non-parametric tests were applied for statistical analysis. Differences were considered statistically significant when p < 0.05.

## Discussion

Results from the present study show that genital HPV infection is commonly present in a population of male partners of infertile couples from Argentina. However, no association between HPV infection and alterations in sperm quality was found. Nonetheless, and up to our knowledge, this is the first study that shows a significant association between HPV and *C. trachomatis* infection in male partners of infertile couples.

The reported prevalence of HPV infection in men varies widely worldwide and it has been described to be 2–31% in the general population and 10–35.7% in men with unexplained infertility^22^. Analysis of the literature indicates a higher percentage of infection in infertile men compared with either the general population or fertile men^17,18^. The prevalence of HPV infection in male partners of infertile couples reported herein (27.4%) is slightly higher than the prevalence already reported in other regions or countries around the world^19,23,27^, with the exception of Golob et al*.*, who reported a prevalence of 37% in the external genitalia and 13% in semen samples from male partners of infertile couples^15^. In Córdoba, Argentina an overall prevalence of 8.3% has been reported in semen samples from men from general population^28^. Thus, our results show higher prevalence of HPV genital tract infection in male partners of infertile couples with respect to men from the general population. The prevalence of male genital HPV infection appears to vary substantially between regions and countries, with the lowest reported in asian men^29^. In addition, the prevalence of genital HPV infection in men from Brazil, Mexico, and USA has also showed significant differences that could be explained by many factors such as diverse sexual behaviors and practices and, ethnic-specific factors in the frequency of germline variations in various immune regulating genes. All these findings highlight the importance to study the prevalence of STI in different parts of the world^30^. Regarding HPV genotype distribution, our results showed a strikingly elevated prevalence of HPV6, followed by HPV16, HPV18, HPV31 and HPV53. Although other authors found HPV16 as the most prevalent in semen samples^19,31^, our results agree with those published by Shigehara et al., who demonstrated that HPV6 was the most common HPV type in the urethral swabs obtained from men with urethritis^32^. It is important to take into account that our study used DNA extracted from a mixed sample composed of urethral swabs and semen to detect HPV. This approach enhances the possibility of detecting HPV infections in the lower and upper male genital tract, which could be related to the higher prevalence of HPV infection detected and to the particular HPV genotype distribution found. It has been reported that sampling different anatomical sites and/or using different combinations of samples increment the chances of detecting HPV infection since it often occurs at multiple anatomic sites within the same individual, being mainly detectable in the skin of external genitalia or in urethra swabs than in semen^14,15,32^. However, a main limitation of our study is that HPV detection was performed in combined specimens from different anatomical sites that were sampled together and not separately. Therefore, we could not assess the individual contribution of each sample to the total prevalence and possible differences in genotype distribution according to the sample analyzed. Nevertheless, bibliography indicate that different locations in the same patient often shares the same HPV genotype^15,33^. Indeed, when HPV genotypes were analyzed in penile skin, semen and vas deferens from the same patient, high concordance of specific HPV genotypes were found in penile skin and semen samples^33^. In addition, Golob et al. reported a higher prevalence of HPV infection in external genitalia than in semen specimens. Moreover, they found a high concordance in HPV types found in both samples^15^.

The relationship between HPV male urogenital infection and alterations in sperm quality is a matter of debate^18,22,34^. Some studies have shown that HPV male urogenital infection associates with reduced sperm motility^23,24,31,35^. On the contrary, and consistent with our findings, other reports have shown no association between HPV male urogenital infection and sperm quality alterations^15,25,27,36^. A possible explanation for this discrepancy may be the anatomical location of HPV infection. Using different experimental approaches, Foresta et al. showed that reduced sperm motility was found in HPV-infected patients. Interestingly, HPV attached to the head of spermatozoa was found in a significant fraction of these patients, suggesting that a direct interaction of HPV with spermatozoa could impair sperm motility^35^. Therefore, it is likely that reports in which impaired sperm motility was found in HPV-infected patients included patients in whom HPV infection is present in the upper and/or lower male urogenital tract, which would enhance the chances of HPV-ejaculated sperm interaction, rather than in external genitalia. Besides, it could be speculated that positivity in men could be a temporary phenomenon, not affecting semen quality. However, it seems that male infection is not short lived since follow-up testing to check HPV clearance indicated that 63.9% and 14.7% of infertile men were still HPV positive in semen after 12 and 24 months, respectively^13^. In addition, another important aspect that could have contributed to that controversial issue is the putative role of co-infections. Indeed, in most reports in which alterations in semen quality were shown in HPV infected patients, no other possible concomitant uropathogens infections were assessed, thus neglecting their possible contribution^13,23,24^.

HPV co-infection with other STI was more thoroughly investigated in females. de Abreu et al. have shown that women with urogenital co-infection of HR-HPV with *C. trachomatis* are at higher risk of having abnormal cervical cytology^37^. Regarding *C. trachomatis* and HPV co-infection of the male genital tract, available evidence comes from studies focused on the potential oncogenic risk of these associated infections in specific groups of patients^38,39^. HPV co-infection with other STI, such as *Neisseria gonorrhoeae*, *C. trachomatis*, *Mycoplasma* spp. and *Ureaplasma* spp. has been previously described in men with urethritis^32^. STI prevalence reported in the present cohort of male partners of infertile couples were similar to those found in a recent study from our country^40^. Certainly, the prevalence of *C. trachomatis* and *Ureaplasma* spp urogenital infection in a large cohort of infertile patients were similar to that found herein. Nonetheless and up to our knowledge, we are reporting for the first time a tight association between HPV and *C. trachomatis* infections in male partners of infertile couples. Although this finding should be interpreted with caution because of low number of *C. trachomatis* positive cases found in our cohort, bivariate and multivariate analysis confirmed a significant association between HPV and *C. trachomatis* infections. This evidence highlights the importance of including the screening of urogenital infections in the initial diagnostic workup of the infertile couple.

Microorganisms causing chronic inflammatory diseases, such as *C. trachomatis*, have been investigated as associated possible risk factors for HPV transmission and persistence cooperating in the carcinogenesis process and may be with fertility alterations, especially in the female genital tract. The effect of HPV and *C. trachomatis* co-infections was also analyzed in men with chronic prostatitis-related symptoms showing that men with HPV and *C. trachomatis* co-infection have reduced sperm motility and lower counts of sperm with normal morphological forms^41^. When we analyzed sperm quality parameters in patients co-infected with HPV and the other uropathogens under study, no significant differences were observed in sperm concentration, viability, motility or normal morphology with respect to patients infected with HPV alone. Unfortunately, sperm quality analysis in patients co-infected with HPV and *C. trachomatis* with respect to patients infected with *C. trachomatis* or HPV alone could not be performed due to the low number of cases of the sole *C. trachomatis* infection. It has been shown that both, HPV and *C. trachomatis*, can attach to human spermatozoa in vitro with a subsequent increase in sperm DNA damage^31,42^. Beyond dissimilar results regarding whether HPV presence in semen affects sperm quality or not, there is growing evidence showing that sub-fertile women receiving inseminations with HPV + semen show reduced clinical pregnancy rates^43^. Moreover, it has been shown that prophylactic therapy with HPV vaccination improves the reproductive outcome in infertile males with HPV semen infection^44^. Although no differences were found in the semen quality of patients studied herein, is possible that HPV and/or *C. trachomatis* transmission to the female partner could have consequences on the couple's fertility.

In conclusion, our findings revealed a high prevalence of HPV urogenital infection among male partners of infertile couples from Argentina, and a tight association between HPV and *C. trachomatis* infections. Nevertheless, no significant semen quality alterations were observed in either patient infected with HPV alone or patients infected with HPV and other common uropathogens. However, since men provide a reservoir for continued transmission of *C. trachomatis* and HPV to women, our study highlights the need of including routine screening and monitoring of these urogenital infections in male partners of infertile couples.

## Patients and methods

### Study population and clinical specimens

One hundred and seventeen (n = 117) male partners of couples seeking diagnosis for their infertility attending the Andrology and Reproduction Laboratory (LAR), Cordoba, Argentina were included in the study. Eligible men were aged 18 years or older that had semen analysis requested by their physician as part of initial fertility evaluation, after failing to conceive with their partner after one year of unprotected intercourse. Participants who met the following criteria were excluded: varicocele grades 3–4, cryptorchidism, antibiotic treatment within the previous three months and patients receiving chemo/radiotherapy. Following the worldwide-accepted WHO criteria^45^, primary infertility was diagnosed when the couple was unable to conceive a clinical pregnancy after at least 12 months of unprotected intercourse, while secondary infertility refers to couples that achieved a clinical pregnancy at least once in the past but are currently infertile.

The study was carried out in accordance with The Code of Ethics of the World Medical Association (Declaration of Helsinki) standards and the Argentinian legislation for protection of personal data (Law 25,326). The experimental protocol was approved by the Ethics Committee and Internal Review Board (CIEIS) of the Oulton-Romagosa Medical Center, Cordoba, Argentina (code RePIS# 004). Participation in the study was voluntary and participants signed a written informed consent for their inclusion in it. A short questionnaire to inquire about their reproductive health history, their female partners and exposure to different risk factors for infertility was performed. Active smokers were defined as men consuming 1 or more packs of 10 cigarettes per day, whereas non-smokers were men that did not smoke. Semen samples and urethral swabs were obtained from each individual included in the study. Semen samples were collected after 3–5 days of sexual abstinence by masturbation and ejaculation directly into standard sterile containers and delivered to the laboratory within 1 h of collection. Urethral swabs samples were collected by health care professionals, using a Dacron collection swab (DELTALAB, Spain) that was inserted 2 cm into the urethra and rotated for 360 degrees whilst removing it. The samples were placed in sterile tubes containing 1 ml of SPG (0.22 M sucrose, 0.02 M sodium phosphate, 5 mM glutamic acid; 0.2 mm filtered, pH 7.4) until processing. After removing an aliquot of semen to perform semen quality studies, the swab was placed in the paired semen container, mixed and centrifugated 10 min at 500 × *g* to obtain the pellet.

### HPV detection and genotyping

The detection of HPV and other uropathogens was performed by polymerase chain reaction (PCR) in DNA purified from the pellet obtained from each patient as described above. Total DNA was extracted using an AccuPrep Genomic DNA Extraction Kit (BIONEER Corp., Republic of Korea) according to the manufacturer’s instructions and subsequently retaining the genomic DNA at 20 °C until processing. HPV was detected by PCR using the sense primer MY11 (5’- GCMCAGGGWCATAAYAATGG -3’) and the antisense primer MY09 (5’-CGTCCMAARGGAWACTGATC-3`), which amplify the L1 region of the viral genome. The reaction mixture consisted of 2.5 mM of each dNTP, 1 U of GoTaq DNA Polymerase (Promega Corp.), 0.6 mM of MgCl_2_, 25 mM of each primer and 5uL of extracted DNA for a final volume of 25 μL. Amplifications were performed in a PCR thermal cycler (C1000 Touch Thermal Cycler, Bio-Rad Laboratories Inc) with an initial denaturation step of 94 °C for 3 min, followed by 35 cycles of denaturation at 94 °C for 1 min, annealing at 55 °C for 1 min, extension for 1 min at 72 °C, and a final extension step of 5 min at 72 °C. PCR products were electrophoresed on a 1.5% agarose gel, stained with 1 mg/mL ECO-Gel 20,000 × Highway dye (INBIO HIGHWAY SA, Argentina), at 95 V for 30 min in TBE buffer (45 mM Tris–borate, 1 mM EDTA, pH 8.0). A 100 bp marker (Invitrogen, Carlsbad, CA, USA) was used as a size standard. A DNA-free sample was used as a negative control and an HPV-positive sample as a positive control. The amplified DNA fragments were visualized on a transilluminator (UVP-BioDoc It, Fisher Scientific) with UV light. The samples showing specific amplification were further analyzed by HPV genotyping. Co-amplification of the human *β*-globin gene was performed as an internal control using the sense primer GH20 (5’ -GAAGAGCCAAGGACAGGTAC-3’) and the antisense primer PC04 (5’ -CAACTTCATCCACGTTCACC-3’) under the same amplification conditions as for HPV. HPV genotyping was performed by PCR-Restriction Fragment Length Polymorphism (PCR–RFLP) analysis as previously described^46,47^. In brief, PCR amplicons were subjected to digestion with 7 restriction enzymes (Bam HI, Dde I, Hae III, Hinf I, Pst I, Rsa I y Sau IIIA) and subsequently were subjected to electrophoretic analysis on a 2% agarose gel at 60 V for 60 min in 1 × TBE buffer. A 100 bp marker (Invitrogen, Carlsbad, CA, USA) was used as a molecular size standard. After electrophoresis, the size of each fragment was determined. Genotypes were assigned after comparing the patterns of molecular weights of fragments for each HPV genotype^46,47^. A total of 71 different HPV genotypes with different associated oncogenic potential could be detected by this methodology: 36 low risk genotypes, 20 high risk genotypes, 10 intermediate risk genotypes (probably carcinogenic) and 5 genotypes of still unknown oncogenic potential.

### Detection of other common uropathogens

The search for *C. trachomatis*, HSV1, HSV2, *M. hominis, U. urealyticum* and other common bacteria was also performed in clinical samples from individuals enrolled in the study. The presence of *Escherichia coli*, *Enterococcus faecalis, Proteus mirabilis* and other common bacteria was analyzed by conventional bacteriologic culture (Nutrient, MacConkey, Chocolate and Blood agar) after 72 h incubation in 5% CO_2_ and aerobic conditions at 37 °C. The presence of *M. hominis* and *U. urealyticum* in semen samples was assessed by the commercially available MYCOFAST Screening RevolutioN colorimetric test (cat 00063, ELITech Group) following the manufacturer's instructions. The presence of *C. trachomatis*, HSV1 and HSV2 was analyzed by PCR in total DNA purified from a mix of urethral swab and a fraction of the semen pellet from each patient, as detailed above. *C. trachomatis* was assessed by amplification of the *ompA* gene using the primers SeroA1, SeroA2, pCTM3, and of the cryptic plasmid gene using the CTP1 and CTP2 primers, as previously described^48^. To assess the presence of HSV1 and HSV2, a multiplex PCR with the primers CTGTGGTGTTTTTGGCATCA (sense) / GGTTGTGGAGGAGACGTTG (antisense) for HSV1 and CATGGGGCGTTTGACCTC (sense) / TACACAGTGATCGGGATGCT (antisense) for HSV2 was performed^49^.

### Semen analysis

Semen analysis was performed according to the WHO 2010 Semen Analysis Manual (fifth edition)^45^, with some modifications. Sperm concentration, progressive motility (PR) and non-progressive motility (NPR) motility were assessed within 1 h after ejaculation. Sperm viability was analyzed using eosin Y (Sigma-Aldrich) staining. Sperm morphology was evaluated by the Papanicolaou technique and according to Kruger's strict criteria. The concentration of round cells was evaluated using the Makler counting chamber (Sefi-Medical Instrument, Haifa, Israel) and peroxidase-positive cells were quantified among round cells using a previously described cytochemical assay^50^. In brief, 20 µl of working solution (containing benzidine and H_2_0_2_) were mixed with 20 µl of liquefied ejaculate, incubated for 5 min at room temperature and counted at 400X magnification on a phase-contrast microscope^50^.

### Statistical analysis

Statistical analysis was performed using the GraphPad Prism version 7.0. (GraphPad Software, La Jolla, CA, USA https://www.graphpad.com/scientific-software/prism/) and IBM SPSS Statistics for Windows, version 25.0 (IBM, Armonk, NY, USA https://www.ibm.com/products/spss-statistics). Data distribution was assessed by the Shapiro–Wilk test. Mann–Whitney or Kruskal–Wallis non-parametric tests were applied for statistical analysis followed by Dunn's multiple comparison test in the latter case. Data are shown as mean ± SD. For nominal data, Pearson's Chi-square test or Fisher's Exact Test, were first performed on a two-way contingency table (n = 117). Fisher's exact test was performed for small samples size. Subsequently, a bivariate and a multivariate logistic regression analysis was carried out to determine the associations between demographic, clinical and co-infection factors and HPV infection. Odds ratios (OR) measure with 95% confidence intervals (95% CIs) were calculated. The subjects with one or more missing data included in the analysis were excluded (n = 16), leaving an analysis group of 101 individuals. Differences were considered statistically significant when *p* < 0.05.

## Supplementary Information

Supplementary Information 1.

Supplementary Information 2.
