# Supplementary figures and images for: Association between Human Papillomavirus and Chlamydia trachomatis genital infections in male partners of infertile couples
Source: Sci Rep. 2021 Oct 7;11:19924. doi: 10.1038/s41598-021-99279-9 (PMC8497555; doi:10.1038/s41598-021-99279-9)

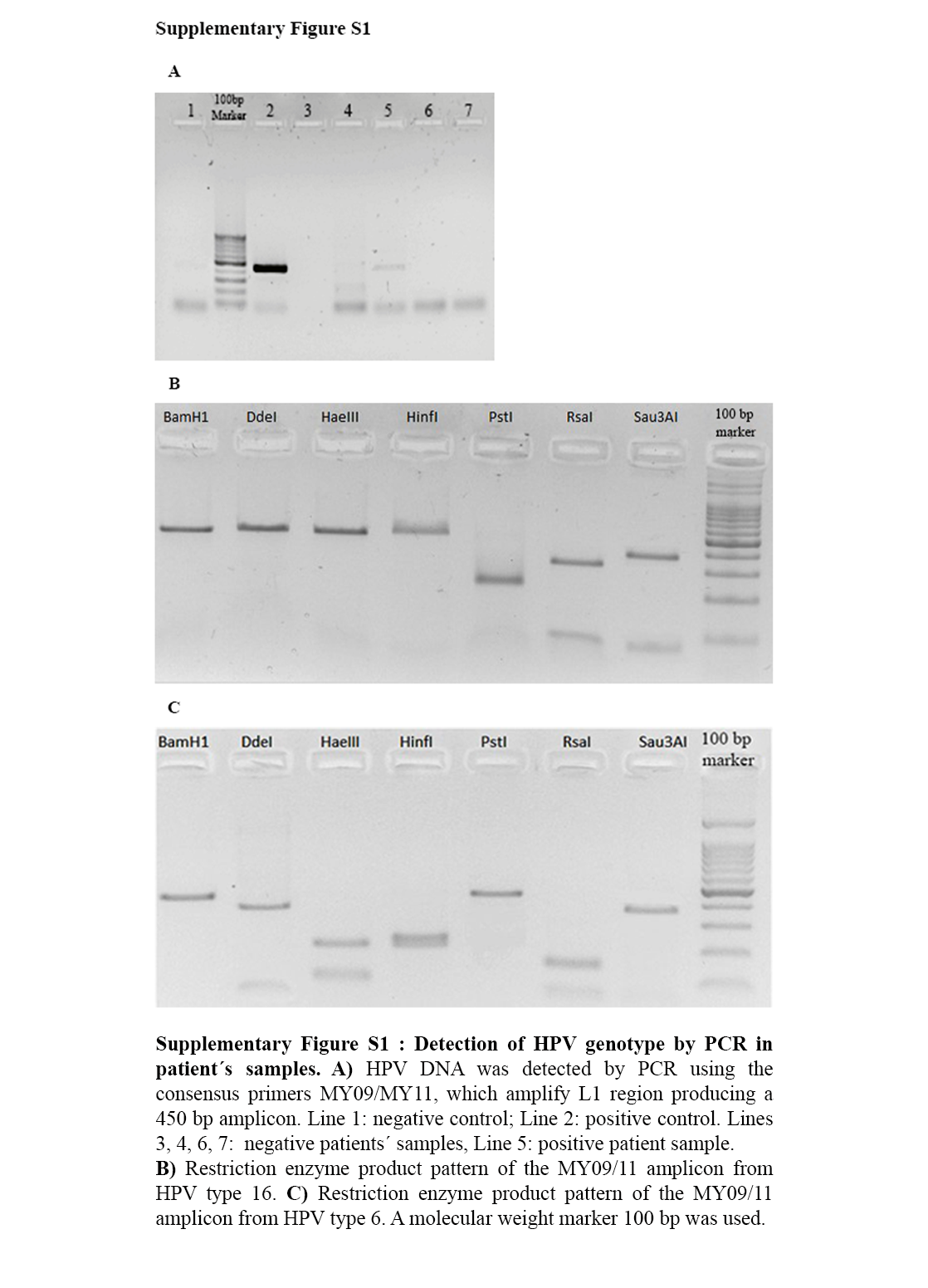

Supplement: Supplementary file 1 — Supplementary Information 1. [file 41598_2021_99279_MOESM1_ESM.tif]
