# Supplementary material for: Association between Human Papillomavirus and Chlamydia trachomatis genital infections in male partners of infertile couples
Source: Sci Rep. 2021 Oct 7;11:19924. doi: 10.1038/s41598-021-99279-9 (PMC8497555; doi:10.1038/s41598-021-99279-9)
Supplement: Supplementary file 2 — Supplementary Information 2. [file 41598_2021_99279_MOESM2_ESM.pdf]

Supplementary Table S1: Bivariate and Multivariate analyses of risk factors for detection of HPV infection

Variables	n	HPV infection		Bivariate analysis			Multivariate analysis		
		Positive	Negative	Odds Ratio	95% CI	*p*	Odds Ratio	95% CI	*p*
**Number of individuals included**	101	23	78						
**Age (years)**									
< 30	4	2	2	2.57	0.30-21.98	0.388	11.24	0.66-190.07	0.094
30-35	37	7	30	0.60	0.18-1.99	0.404	1.03	0.20-5.34	0.976
35-40	35	7	28	0.64	0.19-2.14	0.472	0.83	0.17-4.03	0.820
> 40	25	7	18	1.0 (reference)			1.0 (reference)		.
**Partner´s age (years)**									
< 30	16	2	14	0.21	0.03-1.50	0.121	0.24	0.02-3.32	0.289
30-35	46	10	36	0.42	0.10-1.77	0.235	0.71	0.11-4.82	0.729
35-40	29	7	22	0.48	0.10-2.19	0.342	0.88	0.14-5.66	0.894
> 40	10	4	6	1.0 (reference)			1.0 (reference)		
**Type of infertility**									.
Primary	80	18	62	0.93	0.30-2.89	0.899	1.48	0.33-6.57	0.607
Secondary	21	5	16	1.0 (reference)			1.0 (reference)		
**Time without being able to conceive (years)**									
1-3	78	15	63	0.95	0.18-4.95	0.954	2.37	0.19-29.69	0.505
4-6	13	6	7	3.43	0.52-22.80	0.202	9.37	0.60-145.84	0.11
7-10	10	2	8	1.0 (reference)			1.0 (reference)		
**Male partner smoking status**									
smoker	26	6	20	1.02	0.35-2.96	0.966	1.05	0.31-3.54	0.934

non-smoker	75	17	58	1.0 (reference)			1.0 (reference)		
**HSV1**									
Positive	11	2	9	0.73	0.15-3.65	0.702	0.32	0.03-3.40	0.343
Negative	90	21	69	1.0 (reference)			1.0 (reference)		
**HSV2**									
Positive	23	6	17	1.27	0.43-3.71	0.667	0.92	0.20-4.23	0.916
Negative	78	17	61	1.0 (reference)			1.0 (reference)		
C. trachomatis									
Positive	4	3	1	11.55	1.14-117.06	**0.038**	21.07	1.17-378.54	**0.039**
Negative	97	20	77	1.0 (reference)			1.0 (reference)		
M. hominis									
Positive	23	4	19	0.65	0.20-2.16	0.486	0.73	0.18-2.97	0.661
Negative	78	19	59	1.0 (reference)			1.0 (reference)		
U. urealyticum									
Positive	27	6	21	0.96	0.33-2.76	0.937	1.53	0.37-6.35	0.556
Negative	74	27	57	1.0 (reference)			1.0 (reference)		
**Common bacteria**									
Positive	16	1	15	0.19	0.02-1.53	0.119	0.27	0.03-2.57	0.254
Negative	85	22	63	1.0 (reference)			1.0 (reference)		

*Bivariate and Multivariate logistic regression analysis was performed in the SPSS statistical software to determine the associations between demographic, clinical and co-infection factors and HPV infection. Odds Ratios (OR) measure with 95% confidence intervals (95% CI) were calculated. The subjects with one or more missing data included in the analysis were excluded (n= 101). Differences were considered statistically significant when $p < 0.05$.
